# Development of a Three‐Dimensional Bioengineering Technology to Generate Lung Tissue for Personalized Disease Modeling

**DOI:** 10.5966/sctm.2016-0192

**Published:** 2016-09-15

**Authors:** Dan C. Wilkinson, Jackelyn A. Alva‐Ornelas, Jennifer M.S. Sucre, Preethi Vijayaraj, Abdo Durra, Wade Richardson, Steven J. Jonas, Manash K. Paul, Saravanan Karumbayaram, Bruce Dunn, Brigitte N. Gomperts

**Affiliations:** ^1^ Department of Materials Science and Engineering, University of California, Los Angeles, Los Angeles, California, USA; ^2^ David Geffen School of Medicine at the University of California, Los Angeles, Department of Pediatrics, Children's Discovery and Innovation Institute, Los Angeles, California, USA; ^3^ Eli and Edythe Broad Center of Regenerative Medicine and Stem Cell Research, University of California, Los Angeles, Los Angeles, California, USA

**Keywords:** Lung, Tissue engineering, Disease modeling, Three‐dimensional cell culture

## Abstract

Stem cell technologies, especially patient‐specific, induced stem cell pluripotency and directed differentiation, hold great promise for changing the landscape of medical therapies. Proper exploitation of these methods may lead to personalized organ transplants, but to regenerate organs, it is necessary to develop methods for assembling differentiated cells into functional, organ‐level tissues. The generation of three‐dimensional human tissue models also holds potential for medical advances in disease modeling, as full organ functionality may not be necessary to recapitulate disease pathophysiology. This is specifically true of lung diseases where animal models often do not recapitulate human disease. Here, we present a method for the generation of self‐assembled human lung tissue and its potential for disease modeling and drug discovery for lung diseases characterized by progressive and irreversible scarring such as idiopathic pulmonary fibrosis (IPF). Tissue formation occurs because of the overlapping processes of cellular adhesion to multiple alveolar sac templates, bioreactor rotation, and cellular contraction. Addition of transforming growth factor‐β1 to single cell‐type mesenchymal organoids resulted in morphologic scarring typical of that seen in IPF but not in two‐dimensional IPF fibroblast cultures. Furthermore, this lung organoid may be modified to contain multiple lung cell types assembled into the correct anatomical location, thereby allowing cell‐cell contact and recapitulating the lung microenvironment. Our bottom‐up approach for synthesizing patient‐specific lung tissue in a scalable system allows for the development of relevant human lung disease models with the potential for high throughput drug screening to identify targeted therapies. Stem Cells Translational Medicine *2017;6:622–633*

**Significance Statement**

Lung diseases are among the leading causes of morbidity and mortality worldwide. The development of therapies for lung diseases has been hampered by a lack of human disease models. In this study, a method is presented for the generation of self‐assembled human lung tissue containing multiple lung cell types assembled into the correct anatomical location, thereby allowing cell‐cell contact and recapitulation of the lung microenvironment. This bottom‐up approach for synthesizing patient‐specific lung tissue in a scalable system allows for the development of relevant human lung disease models with the potential for high throughput drug screening to identify targeted therapies.

## Introduction

Lung diseases are among the leading causes of morbidity and mortality worldwide and account for many billions of dollars of health care expenditures 1. As the population ages, the burden from chronic lung diseases is expected to increase, with higher morbidity and mortality rates attributable to them 2. Lung diseases include idiopathic pulmonary fibrosis (IPF), chronic obstructive pulmonary disease, acute lung injury, acute respiratory distress syndrome, and bronchopulmonary dysplasia. These conditions are all characterized by abnormalities of the respiratory membrane that limit gas exchange and for which there are no cures 3. Thus, there is a critical need to develop more effective therapies for these respiratory diseases. For example, a major obstacle for the development of therapies for IPF has been a lack of human models, as in vitro and animal in vivo studies do not faithfully reproduce the pathophysiology of the disease 4, 5. There is also a well‐recognized heterogeneity of these lung diseases with wide variability from patient to patient, although no individualized models are available to screen for the best patient‐specific therapy.

Development of tissue‐engineered organoids (organ mimics) may allow for more accurate disease models and provide a deeper insight into disease pathophysiology, thereby allowing for the identification of new therapies 6, 7. For these organoids to function as such, they must replicate the organ's anatomy, contain the organ's native cell types, and, most important, scaffold those cell types into the appropriate microenvironmental niches. One route is to reverse‐engineer the organ by adhering to the design criteria set down by the histology of the tissue and by scaffolding the appropriate cell types in three dimensions.

Several methods for the generation of engineered lung have been previously developed. There are, in general, two approaches for accomplishing engineered lung, mainly differing in the way the method seeks to develop cell phenotype and three‐dimensional (3D) structure. On one hand, scaffold‐centered methods focus on seeding pulmonary cells onto a 3D structure that is chemically and structurally similar to that of native lung extracellular matrix (ECM). Scaffold materials range from decellularized whole‐lung 8, 9 to biodegradable foams 10, 11, gels 12, and beads 13. So far, the most developed of these methods is the decellularization and reseeding of whole lung. Although decellularized lung may offer the most anatomically correct scaffold, it is hampered by a shortage of donated lungs and scaffold immunogenicity. In addition, this method is not amenable to high throughput applications, thereby hindering drug screening efforts. On the other hand, 3D structure may be developed by driving developmental programs in immature pulmonary cell cultures. This is accomplished by a combination of 3D cell culture, growth factor supplementation, and coculture techniques 14, 15, 16, 17, 18, 19, 20, which results in 3D branching morphogenesis characteristic of early lung development. Here, we present a novel method for the scalable generation of hydrogel‐bead based, self‐assembled human lung organoids and their potential for disease modeling and drug discovery for lung diseases such as IPF. Our approach improves on the previously mentioned methods by providing a modular scheme for directly patterning different combinations of cell types in the naturally occurring lung geometry. This lends itself to high throughput generation of identical, patient‐specific lung organoids amenable to clinical translation.

Our approach reproduces the anatomy of distal lung alveolar sacs by scaffolding mesenchymal cells into the interstitial spaces between closely packed, biocompatible hydrogel beads. In organ systems, form and function are highly related as cell‐level functionality depends on the local microenvironmental cues. Therefore, we hypothesized that recapitulation of the mesenchymal compartment of the lung microenvironment would be critical in replicating the conditions necessary for modeling fibroblast‐driven lung diseases. Here, we show that by combining collagen‐functionalized alginate beads and human fibroblasts in a rotational bioreactor, it is possible to form cohesive organoids with a geometry that mimics that of native lung. Organoid formation occurs because of cellular adhesion to the bead surface, cellular proliferation, and contraction. As proof of principle, we generated a model of the progressive scarring observed in IPF by treating fetal lung fibroblast organoids or induced pluripotent stem cell‐derived mesenchymal cell organoids with exogenous transforming growth factor‐β1 (TGF‐β1) and showed that there is a progressive scarring phenotype in the dish that resembles IPF. Finally, multiple other relevant cell types were incorporated into the organoid seeding process, indicating that this tissue generation process is amenable to multiple cell‐type coculture and could be applicable for modeling other lung diseases.

## Materials and Methods

### Alginate Bead Generation and Functionalization

Alginate beads were generated using an electrostatic droplet generator (custom) operated on a 3% medium viscosity alginate solution (Sigma‐Aldrich, St. Louis, MO, https://www.sigmaaldrich.com) at 9,000 V over a bath of 100 mM BaCl^2^ (Sigma‐Aldrich) solution. Bead size distribution was determined using a custom‐built MATLAB algorithm. White light images of the beads under ×5 magnification were displayed and the user defined each bead diameter by clicking on opposite bead edges. A total of 359 beads were imaged resulting in a size distribution of 161 ± 80 μm. The beads (2.5 ml sedimented) were rinsed and allowed to soak in 1 ml of high‐concentration (9.37 mg/ml) rat tail collagen I solution (Corning, Corning, NY, https://www.corning.com) for 6 days at 4°C. After soaking, 2.5 ml of beads were pipetted into a 35‐mm petri dish (Corning) and the excess collagen I was aspirated, and 8 ml of 2 mg/ml dopamine hydrochloride in 50 mM Tris buffer (Sigma‐Aldrich), pH8.5, was added. The dish was sealed with parafilm (Sigma‐Aldrich) and rotated at 16.5 rpm on a laboratory rotisserie (Thermo Fisher Scientific Life Sciences, Waltham, MA, https://www.thermofisher.com) for 1 hour at room temperature. Beads were then rinsed in the previously mentioned Tris buffer and then soaked in experimentally relevant, serum‐free media.

### Human Fetal Lung Cell Isolation and Cell Culture

Human fetal lung fibroblasts (FLFs) were isolated from 18‐ to 20‐week‐old fetal lungs (Advanced Bioscience Resources, Alameda, CA). Tissues were finely minced and dissociated using 1 mg/ml collagenase/dispase (Roche, Basel, Switzerland, http://www.roche.com) and 0.1 mg/ml DNase (Sigma‐Aldrich) with rotation for 45 minutes at 37°C. After washing in media containing 1% fetal bovine serum (FBS), a single‐cell suspension was generated using 100‐ and 40‐μm cell strainers. To remove red blood cells, the suspension was incubated in red blood cell lysis buffer (BD Biosciences, San Jose, CA, http://www.bdbiosciences.com) for 15 minutes at room temperature. Cells were then plated in 6‐well tissue culture plates and cultured in Dulbecco's modified Eagle's medium (DMEM)/F12 medium containing 10% FBS (Corning). Human umbilical vein endothelial cells (HUVECs) and small airway epithelial cells (SAECs) were maintained according to the manufacturer's recommendations (Lonza, Basel, Switzerland, http://www.lonza.com) in endothelial growth medium (EGM)‐2 (Thermo Fisher) and small airway growth medium (SAGM) (Lonza), respectively.

### Generation of Induced Pluripotent Stem Cells From Healthy Adult Lung Samples and Their Spontaneous Differentiation Along the Mesenchymal Lineage

Adult lung biopsies were procured according to the University of California, Los Angeles (UCLA) institutional review board protocol (no. 08‐09‐038‐01), from the UCLA Medical Center at the time of lung transplantation. Lung biopsies were obtained from five healthy adults. The induced pluripotent stem cells (iPSCs) were generated as per established protocols by Karumbayaram et al. 21. Briefly, the punch biopsy samples were rinsed twice in Hanks’ Balanced Salt Solution (Thermo Fisher) and chopped into 1‐mm pieces in 2% animal origin free collagenase solution (Millipore). After 90 minutes of incubation at 37°C in a 5% CO_2_ incubator, the tissue was collected and centrifuged at 300*g* for 5 minutes. The supernatant was aspirated, and the pellet was washed once with 10 ml of Mesenchymal Stem Cell Medium, Chemically Defined (MSCGM‐CD) (Lonza) and centrifuged as described previously. The pellets containing the dissociated cells and tissue clumps were collected in 2 ml of MSCGM‐CD medium and plated on a CELLstart (Thermo Fisher)‐coated dish. Media were changed once every 72 hours until the cell monolayer was 70% confluent. Cells were passaged using TrypLE (Thermo Fisher) and cryopreserved in ProFreeze‐CDM Chemically Defined Freeze Medium (2×) (Lonza) as per the manufacturer's protocol. For the generation of iPSCs, 1 × 10^5^ fibroblast cells were plated in a CELLstart‐coated well of a 6‐well plate in MSCGM‐CD medium and transduced with STEM Cre‐Excisable Constitutive Polycistronic Lentivirus (STEMCAA) (gift from Dr. Darrell Kotton, Boston University, Boston, MA) vector concentrate (7 × 10^6^ TU/ml) in 1 ml of MSCGM‐CD medium containing 10 μg/ml polybrene (Sigma‐Aldrich) and incubated overnight at 37°C in 5% CO_2_ incubator. The next day, media were aspirated, and cells were rinsed 3 times with MSCGM‐CD and cultured for an additional 3 days in the same medium. On the fifth day, cells were replated in 50:50 TeSR2 (StemCell Technologies)/Nutristem (Stemgent Inc., Vancouver, BC, Canada, https://www.stemcell.com) containing 10 ng/ml Recombinant Human FGF‐basic (154 a.a.) (Peprotech, Rocky Hill, NJ, https://www.peprotech.com) in two 6‐cm dishes coated with CELLstart and cultured until iPSC‐like colonies appeared. The colonies were picked mechanically and cultured in CELLstart‐coated dishes [Recombinant Human FGF‐basic (154 a.a.); Peprotech], and they were passaged mechanically using the EZPassage (Thermo Fisher) tool as per the manufacturer's protocol. The colonies were collected by gentle pipetting and transferred to a 15‐ml tube, and they were passaged at the dilution of 1:6 into a new CELLstart‐coated plate (Thermo Fisher). Three independent iPSC lines per lung sample were generated from lung biopsy.

To induce differentiation of iPSCs along the mesenchymal (osteogenic and adipogenic) lineage, iPSCs were dissociated using 1 mg/ml of dispase for 10 minutes and gently scrapped to collect the colonies. The colonies were rinsed twice in DMEM/F12 medium (Thermo Fisher) and then cultured in nonadherent dishes in DMEM/F12 medium supplemented with 10% FBS (Thermo Fisher), 1× GlutaMAX (Thermo Fisher), 10 nM nonessential amino acids (StemCell Technologies), and 0.1 mM monothioglycerol (Sigma‐Aldrich) for the generation of embryoid bodies. After 4 days, the embryoid bodies were collected and plated on gelatinized dishes to allow to adhere and cultured in media containing DMEM/F12 medium supplemented with 10% FBS, 1× GlutaMAX, and 10 nM nonessential amino acids, and the resulting cells were cultured in DMEM with 10% FBS and additives for 3 weeks 21, 22.

### ACTA2‐mCherry iPSC‐Derived Mesenchymal Cell Line Derivation

Lentiviral particles that express mCherry under the control of the *ACTA2* (α‐smooth muscle actin [α‐SMA]) promoter were purchased from GeneCopoeia (catalog no. LPP‐HPRM14109‐LvPM02; Rockville, MD, http://www.genecopoeia.com). iPSC‐derived mesenchymal cells were plated in a 35‐mm dish at a density of 1 × 10^5^ cells. Cells were approximately 80% confluent the next day and were transduced with 8 μl lentivirus (1.15 × 10^8^ TU/ml) in the presence of 2.0 μl polybrene transfection reagent (10 mg/ml; Millipore, Billerica, MA, http://www.emdmillipore.com) in 1.5 ml DMEM/F12. After 3 hours, cells were supplemented with 10% fetal calf serum. Stable clones were selected with puromycin (1.0 mg/ml; Thermo Fisher). Selected cells were expanded in a T25 flask until 80% confluent.

### Lineage‐Dependent Characterization of iPSC‐Derived Mesenchymal Cells

For osteogenic and adipogenic differentiation, iPSC‐derived mesenchymal cells were plated at densities of 4.2 × 10^3^ cells per cm^2^ and 2.1 × 10^4^ cells per cm^2^, respectively in 8‐chamber slides. After 2 days of incubation at which the cells reached 100% and 70% confluence, the cells were cultured for an additional 14 days in osteogenic differentiation medium (catalog no. CCM008; R&D Systems, Minneapolis, MN, https://www.rndsystems.com) or adipogenic differentiation medium (catalog no. CCM011; R&D Systems). Medium was changed every 2 days. Staining with osteocalcin for the presence of calcium deposits was used to assess the osteogenic differentiation of the cells. Similarly, the presence of lipid vacuoles and a positive stain for FABP4 was used to assess adipogenic differentiation of the iPSC‐derived mesenchymal cells.

### Hydroxyproline Assay to Determine Collagen I Content

Hydroxyproline content was used to quantify the amount of collagen on alginate beads. It was measured colorimetrically by a method described previously 23 with modification. On day 0, sample aliquots of 100 μl of alginate beads were combined with 40 μl of 9.37 mg/ml rat tail collagen I solution in a microcentrifuge tube and stored at 4°C. A single sample aliquot was removed daily, in the course of 6 days, and the excess collagen I solution separated from the beads by pipetting and stored in a separate tube. At the end of the experiment, the beads and excess collagen were resuspended in 50 μl of ddH_2_O, after which 100 μl of 12N HCl (Sigma‐Aldrich) was added and the sample was hydrolyzed for 24 hours at 110°C. A total of 10 μl of samples were transferred to a 96‐well plate in triplicate along with a hydroxyproline standard and evaporated to dryness under vacuum. Samples and standards were rehydrated in 10 μl of ddH_2_O, then 20 μl of isopropanol (Sigma‐Aldrich) was added. Ten microliters of oxidation buffer (1 part 7% chloramine T [Sigma‐Aldrich] and 4 parts acetate citrate buffer [pH 6.0 per 100 ml: 5.7 g of sodium acetate (Sigma‐Aldrich), 3.75 g of trisodium citrate (Sigma‐Aldrich), and 0.55 g of citric acid (Sigma‐Aldrich) in 35.5 ml of isopropyl alcohol and distilled water]) were added to the samples and incubated at room temperature for 4 minutes. Then, 130 μl of analytical isopropanol solution (3 parts Ehrlich's reagent [2 g of paradimethylaminobenzaldehyde (Sigma‐Aldrich) in 3 ml of 60% (vol/vol) perchloric acid (Sigma‐Aldrich)] to 13 parts isopropanol) was added to each well and placed at 60°C for 25 minutes. Samples were then cooled to room temperature for 5 minutes, after which 100 μL of isopropyl alcohol was added. Absorbance was measured at 558 nm using a spectrophotometer.

### Bioreactor Loading and Mesenchymal Organoid Formation: High‐Aspect Ratio Vessel Bioreactor

One milliliter of functionalized alginate beads and 4 million FLFs in media were added to the 4‐ml HARV bioreactor vessel (Synthecon, Houston, TX, http://www.synthecon.com) using the built‐in syringe/valve system. The vessel was screwed into the bioreactor base and the beads allowed to settle, without rotation for 10 minutes. After sedimentation, the bioreactor was powered on to 4 rpm. Organoids were allowed to form and mature in the course of 2 days.

### Time‐Lapse Imaging and Analysis of Organoid Formation

Organoid time‐lapse imaging was accomplished by mounting a GoPro Hero 3 camera onto the 4‐ml high‐aspect ratio vessel (HARV) bioreactor. The mount was custom built using polycaprolactone and included a macro lens and two white light emitting diodes. A high‐capacity battery allowed for extended data acquisition. All time‐lapse experiments had the same initial seeding conditions of 1‐ml functionalized beads and 4 million FLFs. The GoPro camera was set to take images at a frequency of 2 Hz. Controlling bead flow in the HARV bioreactor was accomplished by changing the rotational speed of the vessel. To quantify bead flow characteristics, time‐lapse imaging and video were implemented. A GoPro camera and a custom‐built mount were fixed onto the 4‐ml HARV and different rotational speeds were recorded. At a bead volume:vessel volume ratio of (1:4), the rotational speed was varied from 4 rpm to 30 rpm. Bead trajectories were broken down into two distinct flow regimes, mildly correlated flow and highly correlated flow. Highly correlated flow was dominant at low rpm wherein beads traveled as a group, maximizing bead‐bead interaction. Mildly correlated flow occurred at higher rpm wherein beads orbited in epi‐circular paths forming transient clumps. The bead flow transitions from highly correlated to mildly correlated flow at approximately 12 rpm. Organoid formation was monitored under the highly correlated flow regimen using time‐lapse imaging analysis. To characterize the kinetics of organoid formation, images taken at different time points were analyzed to determine the organoid geometric center as a function of time in addition to measuring organoid “height” and “width.” Analysis of time‐lapse data allowed for the tracking of organoid dimensions and quantification of organoid condensation. Analysis was accomplished by identifying organoid corners and edges in each frame, averaging these points, and referencing that position to the center of the bioreactor vessel. By overlaying the velocity vectors onto an image of the organoid from that time set, it was possible to plot out the bulk organoid trajectory at different time points. During organoid formation, the initial bolus of beads began to condense and stiffen. Velocity fluctuations in the organoid increased with time in culture as the bead‐bead bridges contracted. We performed another finite difference derivative to allow for the derivation of the organoid acceleration. If organoid acceleration is coupled with mass, the sum total of forces acting on the organoid may be arrived at. The main contributor to the organoid mass comes from the alginate beads, cells contribute little to overall mass. Organoid stiffness was determined by dividing the displacement in organoid “height” into the magnitude of the force necessary to generate that displacement. Stiffness increased due the fibroblast contraction of the bead‐bead cellular bridges formed as a consequence of organoid compression bringing beads into direct contact with each other.

### Bioreactor Loading and Mesenchymal Organoid Formation: 96‐Well Bioreactor

Aliquots of 100 μl of functionalized alginate beads and 1.5 × 10^5^ FLFs in 100 μl of media (50:50 DMEM/F12; Corning) were added to each well. Beads and cells were gently, but thoroughly, mixed together and the plate inserted into a modified laboratory rotisserie (Thermo Fisher) and rotated about the plate's central axis at 16.5 rpm. Organoid formation occurred in the course of 3 days.

### Inhibition of Contraction in Organoid Formation Using Blebbistatin

Mesenchymal organoids were prepared in the 96‐well plate as previously mentioned and allowed to mature for 3 days (with daily media changes). On day 4, organoids were given media supplemented with 25 µM, 5 µM blebbistatin (Sigma‐Aldrich), or 1% dimethyl sulfoxide control. Supplemented media were changed daily for the next 9 days. Organoids were imaged daily.

### Generation and Quantification of the FLF Organoid Fibrosis Model

FLF mesenchymal organoids were prepared in the 96‐well plate bioreactor as previously mentioned and allowed to mature for 4 days with daily media changes. On day 4, serum‐free media were introduced and maintained for a total of 2 days with daily changes. On days 6 and 7, organoids were given low‐serum control media (1% FBS) or media supplemented with (10 ng/ml TGF‐β1) and incubated. Organoids were imaged daily. On day 8 hours, the organoids were processed for immunostaining or RNA analysis.

### Bioreactor Loading and iPSC‐based Mesenchymal Organoid Formation: Centrifuge‐Based, 96‐Well System

One milliliter of functionalized alginate beads and 4 million iPSC‐based mesenchymal cells in media were added to the 4‐ml HARV bioreactor vessel (Synthecon) using the built‐in syringe/valve system. The vessel was screwed into the bioreactor base and rotated at 16.5 rpm for 1 hour to allow for cellular adhesion to the bead surface. Cell‐coated beads were removed from the HARV bioreactor and aliquots of 100 μl were partitioned into the wells of a 96‐well plate. The plate was then centrifuged at 1,000*g* for 5 minutes to further sediment and pack the beads. A total of 150 μl of media were then added and changed daily. Organoid formation occurred in the course of 2 days.

### ACTA2‐mCherry iPSC‐derived Mesenchymal Organoid Generation and Fibrosis Model Quantification

Reporter line organoids were generated using the previously described, centrifuge‐based organoid formation technique. These organoids were treated with TGF‐β1 (10 ng/ml; Peprotech) following the same time course and media formulation as the previously described FLF‐based fibrosis model. On day 8, organoids were imaged in a Zeiss LSM 700 wherein tiled, confocal *z*‐stacks were collected for control and TGF‐β1‐treated organoids (under identical laser intensity and exposure conditions). These images were exported and MATLAB was used to quantify the total fluorescence signal. After the application of a low‐intensity threshold, the signal was summed over the *z*‐stacks to provide an overall fluorescence signal. Three‐dimensional rendering of the *z*‐stacks was performed using the Zeiss Zen software.

### Bioreactor Loading and Multicellular Organoid Formation: 96‐Well Bioreactor

Aliquots of 100 μl of functionalized alginate beads and 1.5 × 10^5^ SAECs in 30 ml of SAGM media were added to each well. Beads and cells were gently mixed together and the plate inserted into a modified laboratory rotisserie (Thermo Fisher) and rotated about the plate's central axis at 16.5 rpm. After 1 hour, organoids were observed under a white light microscope to verify cellular adhesion. The excess media were pipetted off and a combination of FLFs and HUVECs (1.5 × 10^5^ cells each) in a volume of 100 μl was added to each well using a 50:50 mixture of SAGM and EGM‐2 media. The cell solution was gently mixed in and the plate was returned to the 96‐well bioreactor for further rotation. After 7 days, with daily media changes of 150 μl, organoids were processed for immunostaining or RNA isolation.

### Immunofluorescence Staining

For whole‐mount staining, organoids were fixed using 4% paraformaldehyde (Thermo Fisher) in Tris‐buffered saline (TBS) for 1 hour at room temperature and permeabilized using 0.1% Triton X‐100 (Sigma‐Aldrich) in TBS for 30 minutes. After blocking in 10% normal goat serum (Thermo Fisher) for 1 hour, organoids were incubated with primary antibodies for 24 hours at 4°C. After washing, organoids were incubated in secondary antibodies (Thermo Fisher) for 2 hours before the addition of 4′,6‐diamidino‐2‐phenylindole (DAPI). For immunofluorescence staining of organoid and lung sections, fixed samples were mounted in Histogel (Thermo Fisher), embedded in paraffin, and sectioned to 4μm. After deparaffinization and rehydration, antigen retrieval was performed using 1 mM ethylenediaminetetraacetic acid in a pressure cooker for 10 minutes. After cooling, slides were permeabilized using 0.2% Triton‐X 100 in PBS, washed in 0.1% Tween‐20 (Sigma‐Aldrich) in TBS, and blocked with Protein Block (Dako, Carpinteria, CA, http://www.dako.com) for 1 hour. After washing, sections were incubated in secondary antibodies and DAPI for 1 hour at room temperature and mounted in Vectashield (Vector Laboratories, Burlingame, CA, https://vectorlabs.com). The following primary antibodies were used: rabbit antivimentin (Bioss, Woburn, MA, https://biossusa.com), mouse anti‐αSMA (Sigma‐Aldrich), mouse anti‐CD31 (Dako), rabbit anti‐pro‐Surfactant Protein B (SPB) and pro‐Surfactant Protein C (SPC) (Seven Hills), mouse antiprocollagen type I (Developmental Studies Hybridoma Bank, Iowa City, Iowa, http://dshb.biology.uiowa.edu), rabbit anti‐T1a (Abcam, Cambridge, United Kingdom, http://www.abcam.com), and rabbit anticytokeratin (wide‐spectrum; Abcam). Confocal imaging was performed using a Zeiss LSM 700. Human adult lung tissues were obtained from healthy donors and procured under institutional review board‐approved protocols at UCLA.

### Real‐Time Polymerase Chain Reaction (Quantitative Polymerase Chain Reaction)

Organoids were processed for RNA using the RNeasy Mini Kit (Qiagen, Hilden, Germany, https://www.qiagen.com) according to the manufacturer's instructions. An on‐column DNase (Qiagen) digestion step was included. cDNA was generated using the TaqMan Reverse Transcription Kit (Applied Biosystems, Foster City, CA, http://www.appliedbiosystems.com) according to manufacturer's instructions. Quantitative polymerase chain reaction (qPCR) was performed using Taq Universal SYBR Green Supermix (Bio‐Rad, Hercules, CA, http://www.bio‐rad.com) on a StepOnePlus PCR system (Applied Biosystems). The following primer sequences were used: α−SMA: Fwd: AAAAGACAGCTACGTGGGTGA, Rev: GCCATGTTCTATCGGGTACTTC; Col1A2: Fwd: GAGCGGTAACAAGGGTGAGC, Rev: CTTCCCCATTAGGGCCTCTC; and vimentin: Fwd: AGTCCACTGAGTACCGGAGAC, Rev: CATTTCACGCATCTGGCGTTC.

## Results

**Figure 1:**
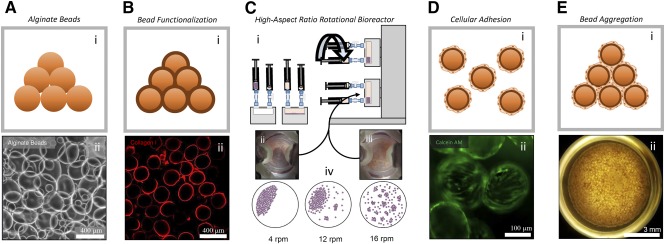
Generation and characterization of 3D pulmonary organoids. Organoids are generated by the agglomeration of cell‐coated alginate beads either in a slowly rotating HARV bioreactor or in a 96‐well plate format. **(Ai):** Alginate bead graphic. **(Aii):** White light micrograph of alginate beads (scale bar = 400 μm). **(Bi):** Graphic showing alginate beads coated with collagen I. **(Bii):** Collagen I immunofluorescence showing a conformal coating of collagen I on the bead surface. Inset, confocal *z*‐stack of a single collagen I‐coated bead (scale bar = 400 μm). **(Ci):** Loading and function of HARV bioreactor. One milliliter of sedimented, functionalized alginate beads were loaded into a 4‐ml vessel. Two million fetal lung fibroblasts were seeded into the vessel. The vessel was attached to the rotary base and rotation initiated. **(Cii):** Time‐lapse image of beads moving together in the 4‐ml HARV bioreactor as a single unit at 4 rpm. **(Ciii):** Image of beads moving independently in the 4‐ml HARV bioreactor at 16 rpm. **(Civ):** Graphical summary of bead flow patterns over several rpm values. **(Di):** Graphic of fetal lung fibroblast‐coated beads after incubation in the HARV bioreactor. **(Dii):** Fluorescence micrograph of calcein AM (viability dye) showing labeled fetal lung fibroblasts evenly coating functionalized beads (scale bar = 100 μm). **(Ei):** Graphic of aggregated, fetal lung fibroblast‐coated beads. **(Eii):** Typical mesenchymal 3D lung organoid generated in the 96‐well bioreactor after 3 days in culture (scale bar = 3 mm). Abbreviations: 3D, three‐dimensional; HARV, high‐aspect‐ratio vessel.

The critical feature in creating lung organoids is the use of functionalized alginate beads that, under rotation in a bioreactor, assemble into a close‐packed architecture that confines cells into the interstitial spaces between beads. Alginate beads 24 161 ± 80 μm (Fig. 1A; supplemental online Fig. 1a) were selected as the template for the alveolar sac because of their biocompatibility and ionotropic crosslinking 25, 26. Bead functionalization is critical as native alginate hydrogels do not support cellular adhesion, a necessary step in the organoid formation process. Bead surface modification was achieved by exploiting a mussel‐inspired adhesion approach allowing for the deposition of a poly(dopamine)/collagen I adlayer 27 (Fig. 1B). A similar modified adhesion technique has been used to modify titanium implants for increased cellular adhesion of MC3T3‐E1 cells 28. Collagen I is a major component of lung ECM, along with other collagens, fibronectin, laminin, elastin, entactin, and proteoglycans 29. Adlayer deposition is a two‐step process of collagen I precipitation 23 (supplemental online Fig. 1b) followed by dopamine polymerization, ultimately allowing for cellular adhesion.

Organoid generation was performed in both HARV bioreactors (Fig. 1C) and a rotating 96‐well plate system. Rotation of functionalized beads with collagen I‐adherent fetal lung fibroblasts in a bioreactor resulted in even coating of the beads by the cells (Fig. 1D). These two approaches offer scalability in both organoid size and number generated, respectively. The process is composed of three steps: (1) loading the bioreactor vessel with functionalized beads and fibroblasts; (2) rotating the vessel to coat the beads with the fibroblasts; and (3) adding additional cell types and further rotating the vessel allowing for organoid aggregation. To improve the throughput potential of the organoid generation method for iPSC‐based drug screening, we combined the HARV and 96‐well approaches. First, beads are coated with fibroblasts in the HARV vessel and then transferred to a 96‐well plate and centrifuged. This combination of bead‐coating and centrifugation leads to sufficient bead‐bead contact for organoid formation and is far more amenable to high throughput organoid generation (Fig. 1E). Once assembled, the organoids remain viable for 2 weeks without degradation.

**Figure 2:**
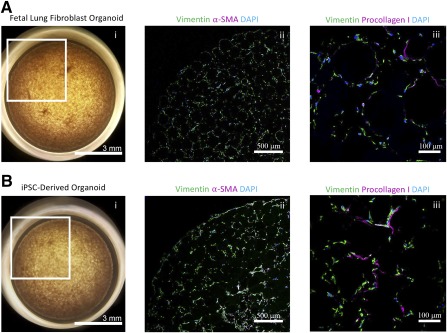
Successful integration of iPSC‐derived fibroblasts into organoid model. **(Ai):** Representative organoid generated using fetal lung fibroblasts. **(Aii, Aiii):** Confocal immunofluorescence micrographs of fetal lung fibroblast organoid sections for vimentin, collagen I, α‐SMA, and DAPI. **(Bi):** Representative organoid generated using iPSC‐derived lung fibroblasts. **(Bii, Biii):** Confocal immunofluorescence micrographs of iPSC‐derived lung fibroblast organoid sections for vimentin, collagen I, α‐SMA, and DAPI. Abbreviations: DAPI, 4′,6‐diamidino‐2‐phenylindole; iPSC, induced pluripotent stem cell; α‐SMA, α‐smooth muscle actin.

We found that the system can be adapted to include any combination of cell types and that iPSC‐derived mesenchymal cells were also amenable to culture in these organoids (Fig. 2). The organoid formation kinetics and cell morphology of the iPSC‐derived organoids was indistinguishable from those derived from fetal lung fibroblasts. Furthermore, the mesenchymal iPSCs demonstrated the ability to be differentiated along several lineages, including osteogenic and adipogenic lineages (supplemental online Fig. 2). Therefore, we were able to demonstrate the ability to personalize this approach for disease modeling and drug discovery.

Organoid formation occurs because of the overlapping processes of cellular adhesion to the bead surface, bead‐bead interactions are caused by bioreactor rotation, and cellular contraction. Bead‐bead interaction is user controlled by altering bioreactor rotational speed, whereas cellular adhesion and contraction are governed by inbuilt cellular machinery. Mesenchymal cells were critical for the formation of structurally robust organoids as seeding other cell types under similar conditions did not allow the organoid to aggregate and form a cohesive tissue (supplemental online Fig. 3). Mesenchymal contraction is essential for proper wound healing and has been implicated as the driving force for organoid condensation in another organoid generation system 30. Although both bioreactor types (HARV and 96‐well) allow for organoid formation, there were some differences in the organoid formation mechanism. Specifically, the HARV system allows for control of organoid formation kinetics and bead flow patterns for a single, large organoid. On the other hand, the 96‐well system achieves organoid formation by offering multiple wells but sacrifices control of bead flow characteristics. Beads in the 96‐well plate fill the entirety of the well bottom precluding the flow patterns achievable in the HARV system. Furthermore, each well in the 96‐well plate is positioned at a fixed distance from the axis of rotation located at the center of the plate. As the radius from the center increases, the centrifugal force experienced by the beads in each well varies from the centermost to the outermost by nearly a factor of 9.3. Yet, despite this variation, organoid formation remained possible in all 96 wells.

**Figure 3:**
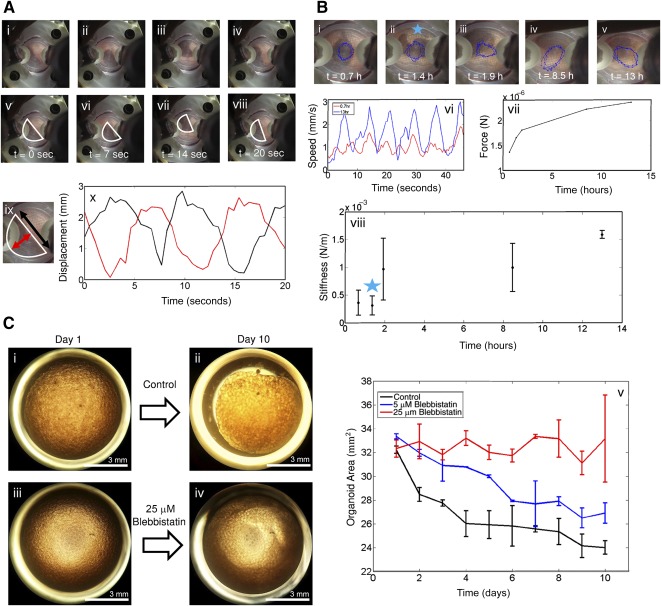
Characterization of the mechanism of organoid formation. **(Ai–Aiv):** Representative images taken of organoid formation after 0.7 hours. **(Av–Aviii):** Organoid position over time is highlighted indicating cyclic deformation with a period of transit of 0.05 Hz. **(Aix):** Red and black arrows indicate user‐tracked dimensions of organoid. **(Ax):** Measured strain versus time plot of indicated organoid dimensions at the 0.7‐hour time point (colors coordinate to dimensions specified in **Aix**). This process increases bead‐bead interactions aiding organoid formation. **(Bi–Bv):** Images of organoids at various time points during organoid formation. Superimposed is a blue track comprised of velocity vectors arrived at during the tracking process. At the 1.5‐hour time point, the organoid developed a defect, artificially increasing the measured strain for that time sequence. This rip was repaired shortly after, indicating the active role fibroblasts play in organoid formation. **(Bvi):** Plot of organoid speed over 50 seconds at 2 different time series. **(Bvii):** Plot of observed force applied to organoid during the 13‐hour period. This increase in force is caused by increased organoid elasticity; as the organoid stiffens, less energy is dissipated by bead‐bead friction and the organoid speeds up. **(Bviii):** Organoid stiffness versus time plot. **(Ci–Civ):** Effect of blebbistatin, a myosin II heavy chain phosphorylation inhibitor, on organoid contraction. Organoid contraction either slowed or was completely inhibited by adding increasing amounts of blebbistatin to culture media (scale bars = 3mm). **(Cv):** Plot of organoid area versus time at different concentrations of blebbistatin.

To identify the underlying mechanisms of HARV bioreactor‐generated mesenchymal organoids, we sought to quantify and characterize the bead flow characteristics and organoid formation kinetics. Video imaging (supplemental online Video 1) was used to identify flow regimes that maximized bead‐bead interactions (supplemental online Fig. 4). Time‐lapse imaging (supplemental online Videos 2, 3; Fig. 3A) and subsequent image analysis was performed under the identified flow regimen to identify organoid position (supplemental online Fig. 5) and geometry (Fig. 3A) over representative 50 second periods of the total 13 hours of recorded organoid formation. The resulting analysis allowed for the characterization of cyclic organoid deformation (Fig. 3A). In addition, it was possible to plot out organoid trajectory and resulting speed with time (Fig. 3B; supplemental online Video 3). As the organoid matures, its average speed through the vessel increases by nearly twofold (Fig. 3B). These measurements were then used to compute the average force experienced by the organoid (Fig. 3B) and, finally, the increase in organoid stiffness with time (Fig. 3B). This increase in stiffness cannot be attributed to the bead flow alone, a cellular mechanism is necessary to further explain this observation.

We then sought to identify the cellular mechanisms of organoid contraction and the observed increase in stiffness. We found that formation of cohesive, dense organoids was only possible if mesenchymal cells were added to the cell seeding process. Other cell types, including HUVECs and SAECs, when seeded alone with functionalized beads, were observed to coat the beads and form loosely associated agglomerations, but contraction and densification were never observed. These organoids remained a fragile, loosely associated bead clump whose removal from the bioreactor chamber inevitably resulted in structural collapse (supplemental online Fig. 3). Therefore, it became apparent that the mesenchymal cells were critical for lung organoid formation, especially in their ability to form bead‐bead bridges and for contraction (supplemental online Fig. 6). To test the effect of mesenchymal contraction, blebbistatin, a myosin II heavy chain phosphorylation inhibitor, was added to the culture media during organoid formation in both the HARV and 96‐well bioreactor. Time lapse imaging and subsequent analysis of both 96‐well‐generated organoids (Fig. 3C) and HARV‐generated organoids (supplemental online Video 4) showed blebbistatin either slowed or completely inhibited contraction of the organoid.

The ability to scale the generation (easily increase the number of organoids generated) of mesenchymal organoids offers a unique opportunity for studying difficult to model diseases such as IPF. IPF is a devastating scarring lung disease characterized by mesenchymal proliferation, contraction, and ECM remodeling 31, 32. The pathogenesis of IPF is unknown, although it is believed to be a fibroblast‐driven, complex, multifactorial disease arising from an abnormal wound healing response to multiple microscopic injuries 5 and asymmetric stress distributions caused by heterogeneous alveolar geometries 33. In addition, analysis of IPF patient samples shows transcriptional similarities between IPF and fetal lung development 34, indicating that studies employing immature pulmonary cells may yield relevant results. Regular two‐dimensional (2D) cultures of lung fibroblasts derived from IPF patients do not demonstrate the morphological fibroblastic foci that are classic for the disease 35. In addition, animal models have failed to recapitulate many of the features seen in patients with IPF. Thus, compounds identified to reverse fibrosis in animal models and 2D tissue culture screens have failed to cure humans 4, underscoring the need for a relevant human disease model.

**Figure 4:**
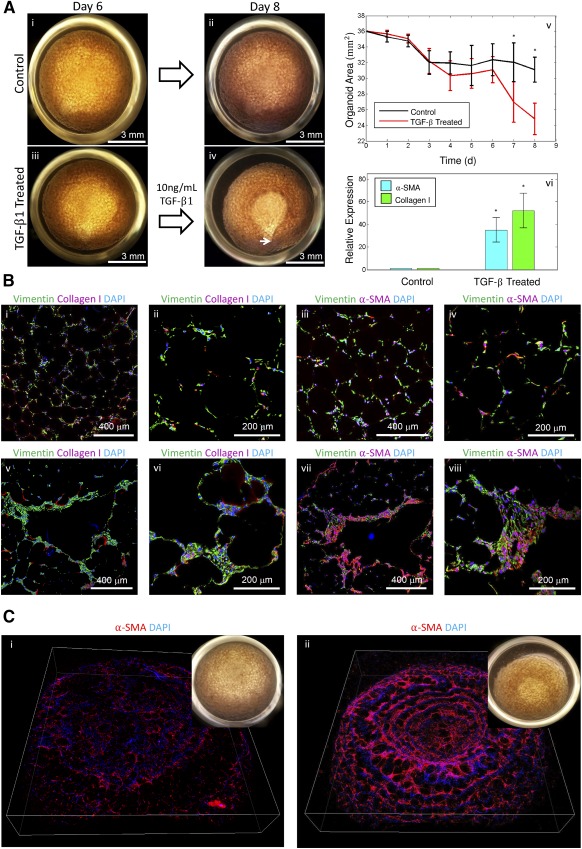
Effect of TGF‐β1 on organoid contraction and development of a fibrotic phenotype. **(Ai, Aii):** Representative control organoid imaged on days 6 and 8 after seeding (scale bar = 3 mm). **(Aiii, Aiv):** Representative organoid treated with TGF‐β1 during the same 2‐day period. The organoid contracted forming a saddle‐like geometry with the focal point near the bottom of the image indicated by an arrow (scale bars = 3 mm). **(Av):** Aggregate analysis of 20 organoids (10 experimental, 10 control) analyzed during the 8‐day experiment. TGF‐β1 was administered on day 6; thereafter, a clear separation between experimental and control organoid contraction was observed; ∗, *p* < .05. **(Avi):** Expression levels of two key genes involved in fibrosis, collagen I and α‐SMA, on treatment with 10 ng/ml TGF‐β1 by quantitative polymerase chain reaction; ∗, *p* < .05. **(Bi–Biv):** Confocal immunofluorescence micrographs of representative control organoid sections for vimentin, collagen I, α‐SMA, and DAPI. **(Bv–Bviii):** Confocal immunofluorescence micrograph of representative TGF‐β1 treated organoid sections for vimentin, collagen I, α‐SMA, and DAPI. Fibrotic areas show increased accumulation of cells that stain positive for collagen I and α‐SMA resembling fibrotic foci, the hallmark of idiopathic pulmonary fibrosis. (Scale bars = 400 μm **[Bi, Biii, Bv, Bvii]**, 200 μm **[Bii, Biv, Bvi, Bviii]**.) **(Ci):** Merged, rotated confocal *z*‐stack of patient, iPSC‐derived, α‐SMA reporter line control organoid. Inset, white light image of organoid. **(Cii):** Merged confocal *z*‐stack of patient, iPSC‐derived, α‐SMA reporter line organoid treated with TGF‐β1. Inset, white light image of organoid showing high degree of contraction. Abbreviations: DAPI, 4′,6‐diamidino‐2‐phenylindole; α‐SMA, α‐smooth muscle actin; TGF, transforming growth factor.

To determine the feasibility of this method in modeling IPF, we decided to examine the effects of exogenous TGF‐β1 on our fetal and iPSC‐derived mesenchymal organoid cultures. We chose to use fetal lung fibroblasts for these studies because of the transcriptional homologies between IPF and fetal lung samples 34. TGF‐β1 is known to play a central role in the development of tissue fibrosis as it causes fibroblasts to differentiate into a myofibroblast phenotype and synthesize and contact ECM 36. Mature fetal lung organoids were treated with exogenous TGF‐β1. Organoid size was monitored daily revealing that treated organoids contracted at a higher rate when compared with untreated organoids (Fig. 4A). Hematoxylin and eosin micrographs indicate that this contraction leads to increased bead packing and overall denser, smaller organoids (supplemental online Fig. 7). qPCR identified an increase in expression of collagen I and α‐SMA in the TGF‐β1‐treated samples when compared with controls (Fig. 4A). Immunofluorescence of sectioned organoids indicated higher levels of collagen I and local patches of α‐SMA, demonstrating activated myofibroblasts, which morphologically resembled the fibroblastic foci that are the hallmarks of IPF (Fig. 4B). Further analysis of the TGF‐β1‐treated fetal lung organoids showed increased numbers of pAKT and Ki‐67 positive cells when compared with control untreated organoids, highlighting the role of TGF‐β1 in fibroblast activation. Specifically, analysis of immunofluorescence micrographs or organoid sections showed 83% ± 6% of all cells in the TGF‐β1‐treated sample were positive for Ki‐67, in comparison with only 3% ± 1% in the control sample (supplemental online Fig. 8).

To perform a successful high throughput screen, it is necessary to develop an assay of a disease model that both faithfully recapitulates the disease and is reproducible over the large number of culture samples. In addition, the characterization and analysis of the assay needs to be done efficiently and reproducibly. With this in mind, we realized that the process of fixation, sectioning, and immunostaining of thousands of organoids would not be technically feasible. We addressed this problem by using a lentivirus to transduce our patient specific, iPSC‐derived mesenchymal cells to express mCherry under the control of an ACTA2 (α‐2 actin, smooth muscle, or α‐SMA) promoter. These cells were used to form organoids and were put through the previously described TGF‐β1 treatment. Live cell, confocal imaging was used to quantify the fluorescence from both control and treated organoids (Fig. 4C). The fluorescence signal was summed over the *z*‐stacks resulting in a 2.5× increase in mCherry in the TGF‐β1‐treated samples when compared with the control. In addition, a difference in organoid contraction commensurate to that measured in fetal lung organoids was observed. We believe that the combination of these two metrics will provide a robust platform for future TGF‐β1‐based drug screening.

**Figure 5:**
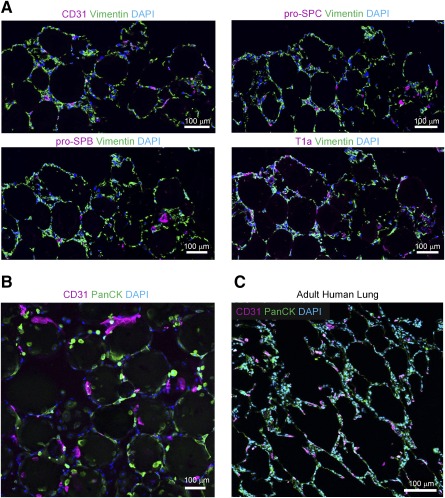
Immunostaining of 3D, multicellular organoids compared with adult human distal lung. **(A):** Confocal micrograph of cross sections of 3D multicellular lung organoids with immunofluorescence for CD31 (HUVECs), vimentin (FLFs), and pro‐SPB and pro‐SPC (type II alveolar epithelial cells) and T1a (type I alveolar epithelial cells; scale bar = 100 μm). **(B):** Confocal micrograph of multicellular 3D lung organoids with immunofluorescence for CD31 (HUVECs) and PanCK (SAECs). FLFs were also seeded. **(C):** Confocal micrograph of a cross‐section of normal adult human lung with immunofluorescence for CD31 (HUVECs) and PanCK (SAECs; scale bar = 100 μm). Abbreviations: 3D, three‐dimensional; DAPI, 4′,6‐diamidino‐2‐phenylindole; FLFs, fetal lung fibroblasts; HUVECs, human umbilical vein endothelial cells; SAECs, small airway epithelial cells; SPB, Surfactant Protein B; SPC, Surfactant Protein C.

Finally, our 3D modeling approach allowed the inclusion of multiple cell types including pulmonary fibroblasts, small airway epithelial cells, and human umbilical vein endothelial cells. These cells retained their classic cellular markers in the organoids as shown with immunofluorescence for CD31 (endothelial cells), pan‐cytokeratin (epithelial cells), vimentin (mesenchymal cells), surfactant protein B and C (type II alveolar cells), and T1a (Pdpn; type I alveolar cells) (Fig. 5A, 5B). In addition, a small fraction of the included small airway epithelial cells were goblet and club cells (<20%; data not shown). We found that the epithelial cells form sheets around the beads and the mesenchymal cells are located in the native lung distribution in the interstitial spaces between the beads (Fig. 5A, 5B). A direct comparison between the engineered multicellular organoids and native human lung alveolar structures show striking similarity in overall morphology of the arrangement of cells around the alveolar sacs and in the interstitium (Fig. 5C). Unfortunately, these organoids did not show spontaneous formation of self‐organizing capillary networks.

## Discussion

The future of tissue engineering lies in the exploitation of the third dimension. Traditional, two‐dimensional cell culture lacks the dynamic complexity and hierarchy of even the simplest of in vivo tissues. This inherently affects the interaction, function and phenotype of the cultured cells and is demonstrated by the fact that most primary cells are difficult to maintain in regular culture. Three‐dimensional systems, on the other hand, mimic the interactions between cells and between cells and their tissue microenvironment by influencing surrounding cell types, scaffold stiffness and degradability, and cell‐cell and cell‐scaffold adhesions, and by establishing cytokine and growth factor gradients. Along these lines, we have developed a method for the generation of distal lung‐like 3D organoids. These organoids were designed to emulate both the architecture and cellular composition of distal lung by scaffolding multiple cells around functionalized hydrogel beads and allowing these beads to interact and condense in a rotational bioreactor. The resulting organoids have a low cellular density with their structure being maintained by the presence of the indwelling alginate beads. These beads serve as a 3D alveolar template, maintaining an opposing force that keeps the interpenetrating cells from contracting into a tight clump. We speculate that the modularity of the beads and cell patterning could be used to model other 3D tissues, which consist of repetitive patterns.

We discovered that organoid formation was not possible without the inclusion of fibroblasts, as organoids that were seeded without fibroblasts failed to contract and lacked the structural integrity of 3D tissue. This observation suggested that organoid formation is analogous to aspects of in vivo wound healing, specifically the tendency for fibroblasts to infiltrate a wound site, lay down collagen I, and contract. Given their mechanism of formation, we hypothesized that these organoids would be prime candidates for modeling fibrotic lung diseases such as IPF. To characterize the organoid response to fibrogenic stimuli, we treated them with exogenous TGF‐β1 and observed increased contraction and expression of collagen 1 and α‐SMA in the treated samples. Although high levels of α‐SMA and collagen I have previously been reported in IPF patient fibroblasts cultured in 2D, we were able to show the morphological and mechanical effects of TGF‐β1 by demonstrating increased contraction and the development of fibroblastic foci within the organoid. Thus, although we only examined one cell type in this IPF disease model, this reductionist approach allowed us to model the pathological hallmark of IPF, which has not been done with human cells in a dish before.

This lung organoid generation method differs from other 3D culture methods in the sense that it exploits the aggregation of many individual cell‐coated scaffold units, alveolar units in this case, to form the extended tissue network. Other scaffold‐based methods require cells to either be perfused into a decellularized lung 8 or to migrate into the interior of a biodegradable foam 37. Our method, on the other hand, ensures that the seeded cells are initially distributed throughout the entire scaffold because each bead is first individually coated with cells. In this case, individual cell coated beads form interbead adhesions and subsequent contraction leads to organoid formation. In addition, the geometry imparted by the agglomeration of the alginate beads introduces a more physiologically relevant scaffold to study the individual cellular mechanics of contraction. Specifically, the curvature of the beads is similar to that of alveolar sacs in vivo. Finally, to fully densify, the organoid fibroblasts must grow into the interstitial spaces between beads. This process of bridging a void, instead of proliferating in a gel, is likely more analogous to the fibrotic processes occurring in vivo.

Takebe et al. 30 have reported that the contraction of mesenchymal stem cells on a soft matrix in the presence of other endothelial and pancreatic cells resulted in a self‐organized organoid that, when transplanted in type 1 diabetic mice, was able to regulate blood glucose levels. Our organoid system is a 3D analog of the work done by Takebe et al. 30 as it also relies on mesenchymal contraction for organoid formation. Unfortunately, our attempts at multicellular culture did not show signs of capillary organization, although it was clear that these cells were able to survive the seeding process and remain viable for 2 weeks in culture.

One of the major advances of our organoid is the fact that it is a high throughput system. This will allow disease modeling in a highly reproducible way, which, together with high throughput confocal scanning, makes drug screening in 3D a reality. The addition of induced pluripotent stem cells into this high throughput 3D system will allow for precision medicine as it is well known that many lung diseases show great heterogeneity among patients. Ultimately, the goal is to develop functional lung organoids that preform gas exchange and could replace damaged patient lungs in an autologous fashion. In the future, these lung organoids may hold great potential to be used as platforms for developing a respiratory membrane with functional vasculature.

## Conclusion

This novel strategy of organoid generation by bioreactor‐assisted self‐assembly allows for fast, easy generation of pulmonary‐like tissues ready for disease modeling. As this method is a bottom‐up synthesis, it is possible to control bead composition, size, stiffness, and functionalization as well as number and type(s) of cells included. These controls may be necessary for the systematic separation of variables necessary to generate the subtleties and heterogeneity of IPF and other lung diseases. In addition, the method is easily scaled in both size and number of organoids bridging the gap between disease modeling and generation of transplant‐ready tissues. In summary, this work introduces a highly reproducible model system to integrate multiple human cell types, including iPSC‐derived cells, in their correct anatomical location to form lung tissue that can be used to model lung diseases and perform high throughput drug screening for precision medicine.

## Author Contributions

D.C.W.: conception and design, collection and/or assembly of data, data analysis and interpretation, manuscript writing, final approval of manuscript; J.A.A.‐O., J.M.S.S., and P.V.: conception and design, collection and/or assembly of data, data analysis and interpretation, manuscript writing; A.D., M.K.P., and S.K.: collection and/or assembly of data; W.R. and S.J.J.: conception and design; collection and/or assembly of data; B.D. and B.N.G.: conception and design, financial support, administrative support, manuscript writing, final approval of manuscript.

## Disclosure of Potential Conflicts of Interest

D.C.W., J.A.A.‐O., J.M.S.S., B.D., and B.N.G have filed for intellectual property rights related to material described in this publication. The other authors indicated no potential conflicts of interest.

## Supporting information

Supporting Information

Supporting Information

Supporting Information

Supporting Information

Supporting Information
