# Supplementary material for: Development of a Three‐Dimensional Bioengineering Technology to Generate Lung Tissue for Personalized Disease Modeling
Source: Stem Cells Transl Med. 2016 Sep 15;6(2):622–33. doi: 10.5966/sctm.2016-0192 (PMC5442826; doi:10.5966/sctm.2016-0192)
Supplement: Supplementary file 1 — Supporting Information [file SCT3-6-622-s001.pdf]

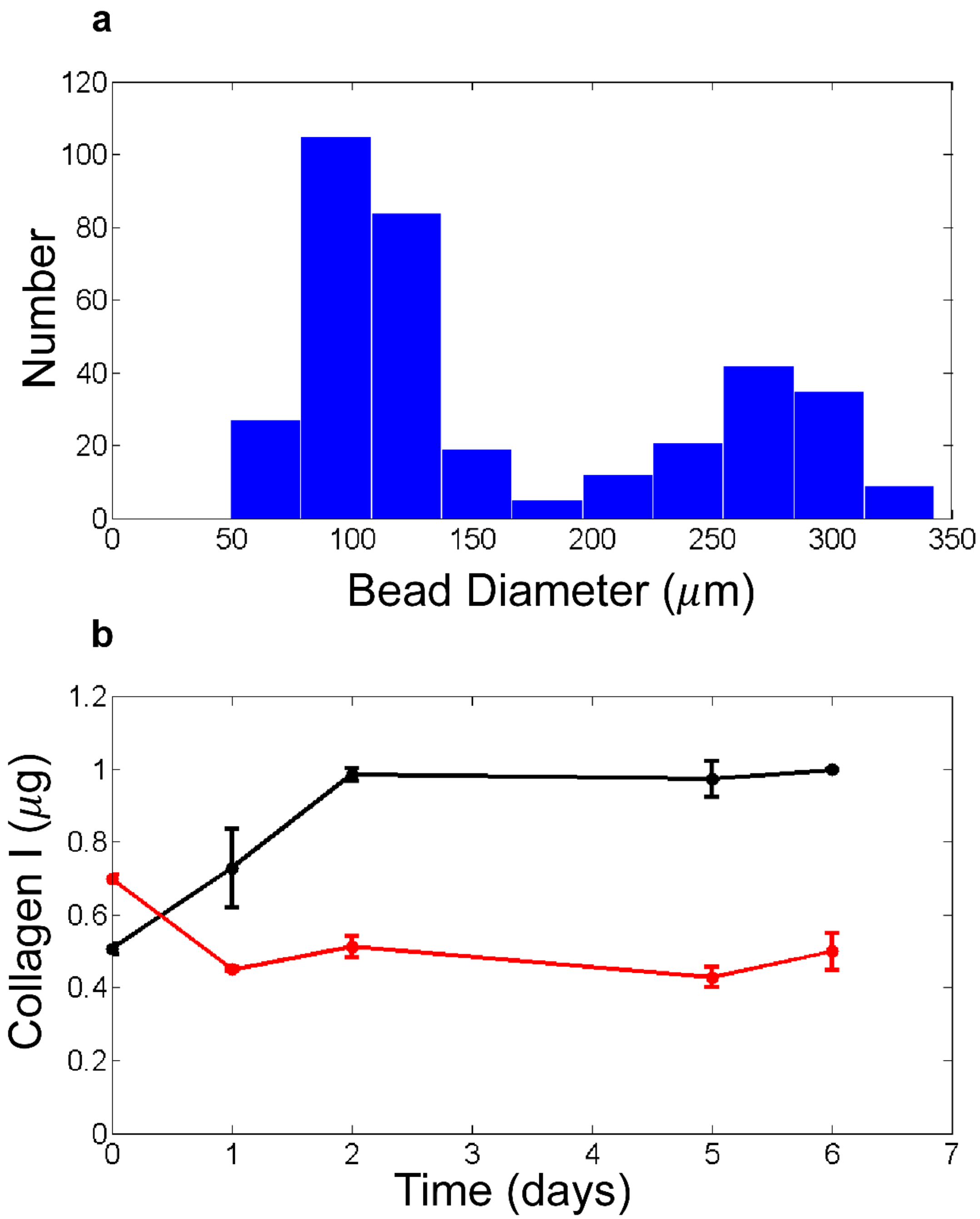

Supplemental Figure 1

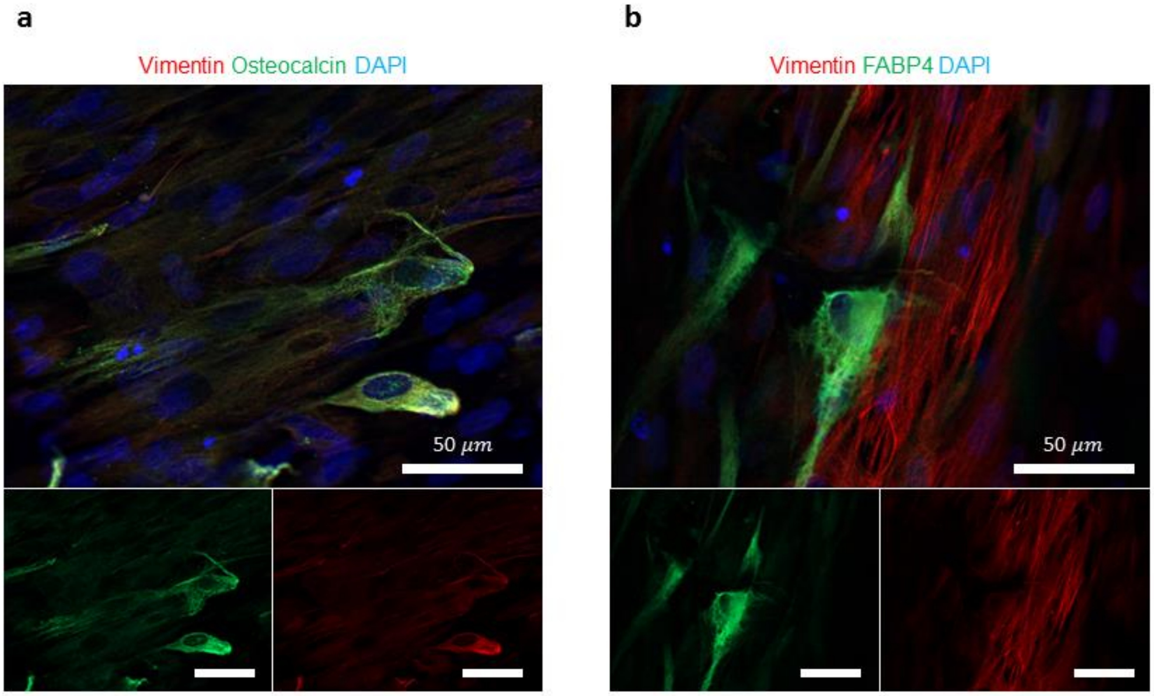

Supplemental Figure 2

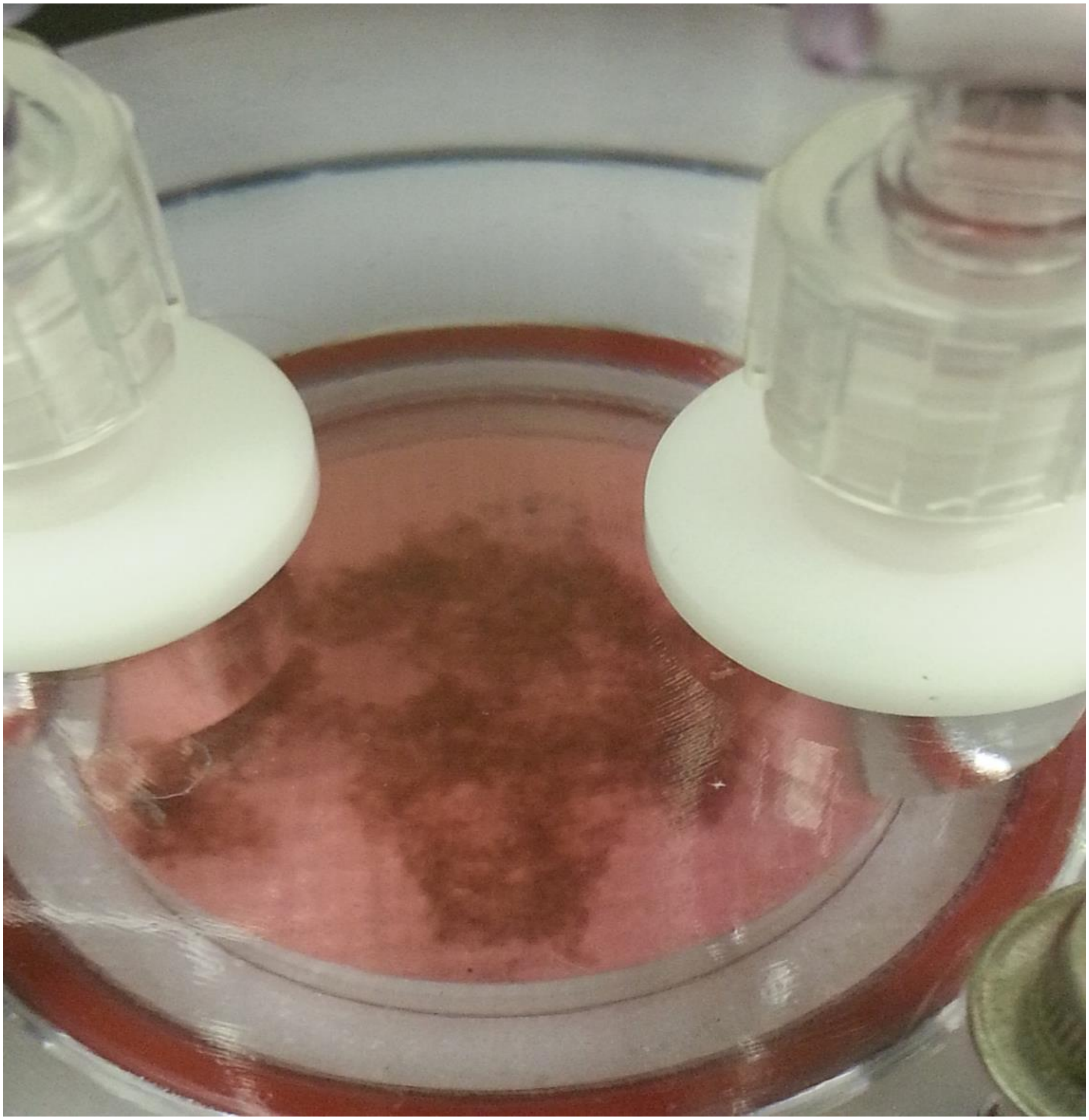

Supplemental Figure 3

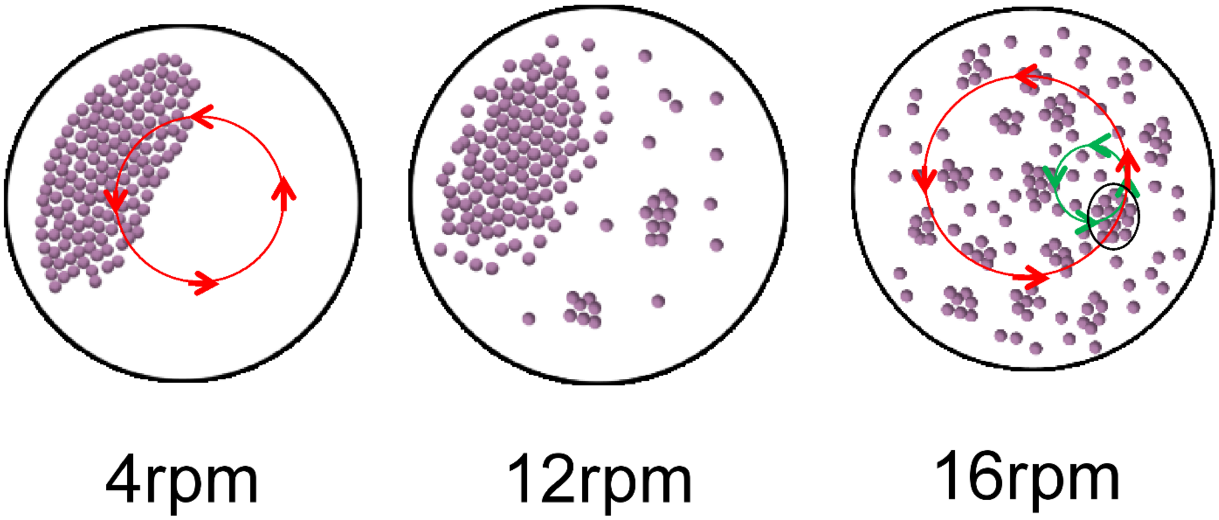

Supplemental Figure 4

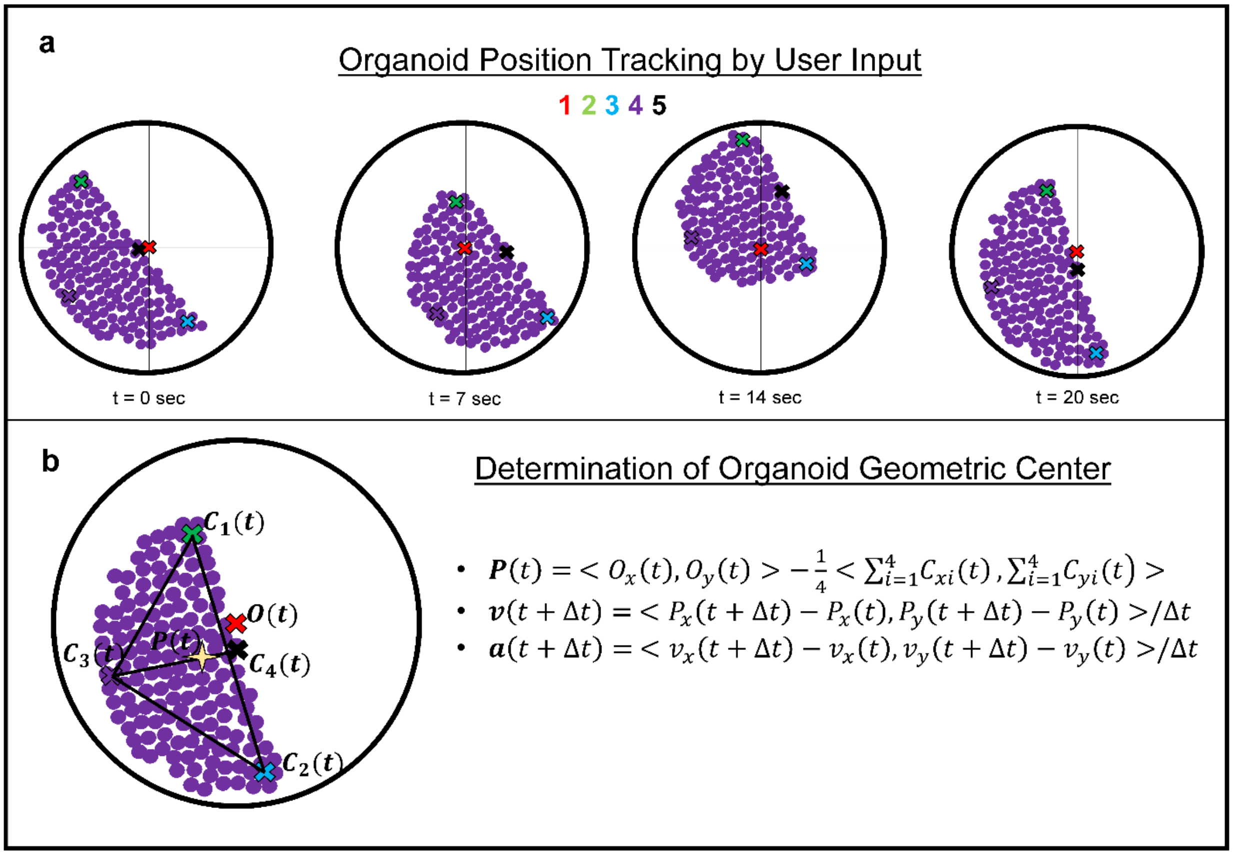

Supplemental Figure 5

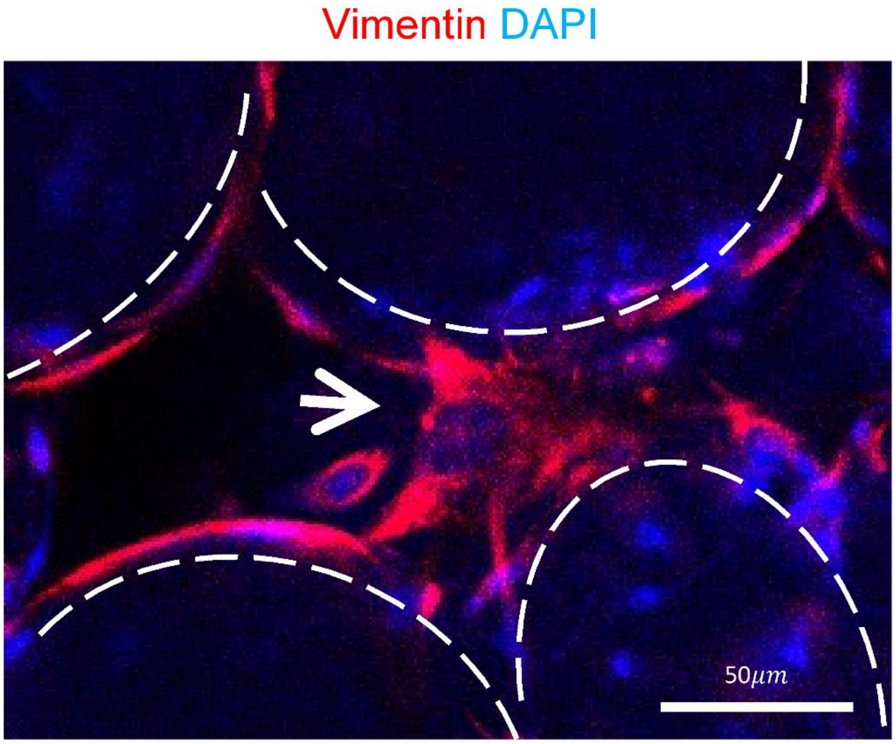

Supplemental Figure 6

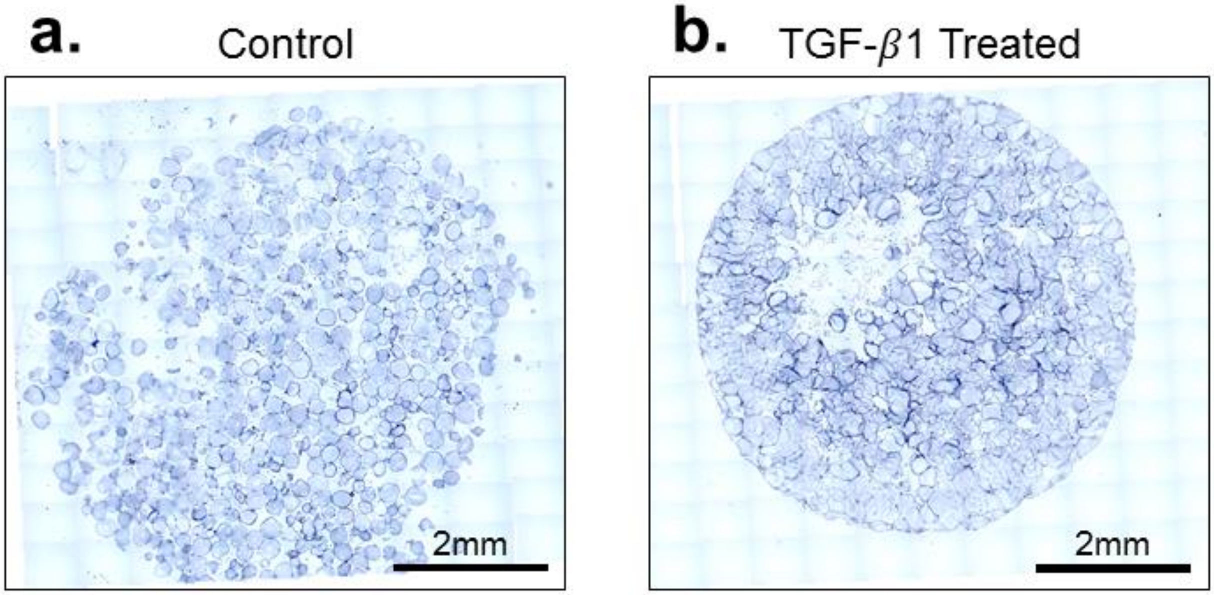

Supplemental Figure 7

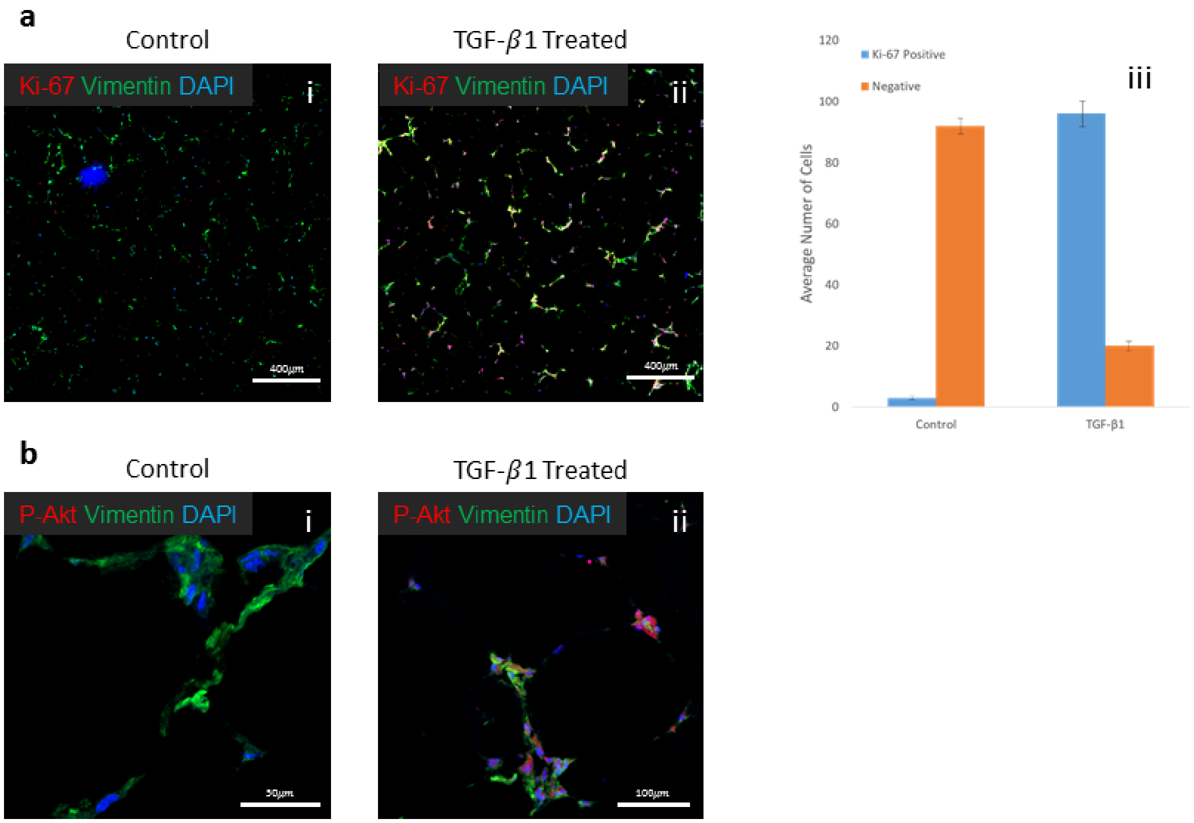

Supplemental Figure 8

SUPPLEMENTAL FIGURE LEGENDS

Supplemental Figure 1. Bead size distribution and collagen I deposition characterization. (**a**) Bead size distribution was quantified by image analysis of white light micrographs using a custom Matlab algorithm. (**b**) Collagen I precipitation during bead incubation was quantified by the colorometric hydroxyproline assay.

Supplemental Figure 2. Immunfluorescent images demonstrating the osteogenic potential (**a**) and adipogenic potential (**b**) of the iPSC derived mesenchymal cells.

Supplemental Figure 3. Example of cell coated beads that failed to aggregate when mesenchymal cells were not included in the bioreactor.

Supplemental Figure 4. Dependence of bead flow pattern on bioreactor rotational speed. At 4rpm beads move as a single bolus under a highly correlated flow condition. As rotational speed increases, at approximately 12rpm, the bead bolus begins to break up. Further increases in rotational speed result in loss of highly correlated flow wherein beads travel in transient clumps in a mildly correlated flow regime. At this point beads travel in epicircular paths.

Supplemental Figure 5. Graphic explanation of the method for organoid tracking. (**a**) Images acquired at a frequency of 2Hz displayed using a custom Matlab program. The five positions

marked per image indicate the organoid edges and the center of the bioreactor vessel. This process was repeated for 100 images (50 seconds) for each data set. (**b**) Organoid geometric center was determined by averaging the position data and subtracting that average from the vessel center.

Supplemental Figure 6. Confocal high power micrograph of vimentin immunostained mesenchymal cells showing cellular bead-bead bridge formation.

Supplemental Figure 7. H&E micrographs of (**a**) control and (**b**) TGF-β1 fetal lung fibroblast organoids. Addition of TGF-β1 to organoid culture caused heightened cellular contraction leading to increased bead packing.

Supplemental Figure 8. Characterization of the TGF-β1 treated organoids. (**a**) Ki67 immunostaining of untreated (**i**) and TGF-β1 treated (**ii**) lung organoids (Scale bar = 400μm) with quantification of proliferating cells in the adjacent bar graph (**iii**). (**b**) P-Akt is present in cells of the TGF-β1 treated organoids but not in any cells in the untreated controls. (**i**) Control organoid. (Scale bar = 50μm). (**ii**) TGF-β1 treated organoid. (Scale bar = 100μm).
